# Jordanian women’s (studying or working in medical fields) awareness in terms of the use of dental imaging during pregnancy

**DOI:** 10.1186/s12903-022-02459-w

**Published:** 2022-09-24

**Authors:** Ammar A. Oglat, Hanan Hasan

**Affiliations:** 1 Department of Medical Imaging, Faculty of Applied Medical Sciences, The Hashemite University, Zarqa, 13133 Jordan; 2 Department of Pathology, Microbiology and Forensic Medicine, School of Medicine, The University of Jordan, Amman, 11942 Jordan

**Keywords:** Dental imaging, Pregnancy, Awareness, Survey, Oral health

## Abstract

**Background:**

The anxiety among pregnant women about the imaging of teeth during pregnancy may have an adverse effect on the oral health of both the mother and the fetus too. This research study was conducted to evaluate women’s knowledge of the utilization of dental imaging during pregnancy.

**Methods:**

In this research, structured questionnaires were distributed electronically through social media. The questionnaires contained questions focused on the women’s (studying or working in medical fields) awareness regarding the ionizing radiation protection that takes place during dental imaging, the safest period for dental imaging, the sort of radiographs that can be required, and the chance of radiation-induced malignant tumor and malformation of the fetal as a result of dental imaging.

**Results:**

Overall, 984 participants completed questionnaires that were analyzed after being received. Most of the participants (n = 637; 64.7%) were < 30 years of age. The greater number of the participants (66.8%) had fair knowledge of dental imaging. 25.4% mentioned that pregnant women are able to do dental imaging during the first trimester. And approximately half of the participants thought that cone-beam computed tomography and panoramic images must not be carried out during pregnancy. Moreover, nearly the same percentage of them also believed that the risk of inborn malformation is high due to dental imaging.

**Conclusions:**

The results refer to a low awareness among people who have medical knowledge regarding dental radiograph protection during pregnancy. This needs to be paid attention to among students, graduates, and workers in medical fields by focusing on the courses and lectures related to dental imaging protection during pregnancy.

**Supplementary Information:**

The online version contains supplementary material available at 10.1186/s12903-022-02459-w.

## Background

The primary tool for discovering, diagnosing, evaluating, and controlling oral abnormalities is dental imaging. Previously, there has been a misconception when it comes to diagnosing the illness of oral diseases via dental imaging and pregnancy. Some organizations, such as the American Congress of Obstetricians and Gynecologists and the American Dental Association, report that it is possible for pregnant women to take dental imaging radiographs during any trimester of their pregnancy period. However, they assume that this is possible only by using and applying the measurements of radiation protection to hold the dose amount as low as reasonably achievable (ALARA) [1–7]. The shortage of knowledge relating to the protection of dental radiographs prevents pregnant women from searching for dental pathologies for treatment. It is important that pregnant women preserve their oral and dental health because the mother’s dental health is related to the fetus’s (offspring’s) dental health [8–15].

Several research studies have shown that both dentists and dental students have a lack of knowledge about diagnostic imaging and radiation protection [16–18]. Medical doctors’ perceptions of the teratogenic risks and impacts of different imaging examinations have been assessed. Research studies have shown that misperceptions are frequent and might have a bad influence on patients’ care [19, 20].

The scarcity of awareness programs in Jordan, lectures about public radiation, and translated articles to the mother tongue language (Arabic) triggered us to evaluate women’s knowledge in terms of the dental imaging radiographs throughout pregnancy. Awareness about radiation protection and safety throughout pregnancy is a crucial part of allowing women to safely search for dental treatment during pregnancy. Therefore, it can be noticed that Jordanian women have fair knowledge and awareness relating to diagnostic dental imaging during the pregnancy period, regardless of their medical background. Moreover, disseminating these results among Jordanian people and universities after they are published will aid in increasing their knowledge level.

An oral radiologist, maxillofacial radiologists, radiologists, medical imaging PhD holders, radiographers, and dentists helped in the development of the questionnaire. This questionnaire was based on the most common misconceptions concerning dental imaging and pregnancy among patients coming to dental clinics.

The main novelty of this study, compared to the previous study done in Jordan in 2020 [18], is that the results from the previous study displayed the general weak knowledge of participants about dental imaging. However, in this study, the main new findings show that the participants who are studying or working in medical fields (medical imaging, medical laboratories, nursing, anesthesia, medicine, etc.) have incomplete (fair) knowledge about dental imaging protection during the pregnancy period. Thus, this is a really serious issue which indicates a shortage of public radiation awareness programs (materials) in all the previous medical fields mentioned above.

## Methods

### Study design and participants

This study was revised and approved by the Institutional Review Board (IRB) at the Faculty of Applied Medical Sciences, The Hashemite University, Zarqa, 13,133, Jordan (#1/4/2021/2022/IRB number). The research followed previous studies’ observational checklist [2]. This cross-sectional self-administration online survey was carried out from September 2021 to December 2021. The modified questionnaires were conducted to assess the awareness and knowledge of women (studying or working in medical fields) relating to the protection measures of dental imaging radiographs throughout pregnancy. A technique of non-prospect snowball sampling was applied to enroll the study population via platforms of social media such as Twitter, Facebook, LinkedIn, Research Gate, WhatsApp, etc. Female participants living in Jordan, aged 18 years or older, were included in this study.

### Sample size

The sample size was calculated by applying the online calculator called the Raosoft sample size calculator, which is available online [21, 22]. This calculation depended on a population size of 496,600 million women. The response distribution is 50%, with a 95% level of confidence and a 5% margin of error. The lower sample size required of participants for this research was 385. However, the last number of enrolled participants was 984 women, to calculate any lost data or non-response averages.

### Questionnaire design

The language of the questionnaire was Arabic, anonymous, and pre-structured. Moreover, it was evaluated by the experts mentioned before in the fields of dentistry and radiology to ensure content validity. After that, it was pre-tested by a selected sample of radiologists and dentists to ensure visibility and face validity. Before starting this study, it was tested with 25 women from the target population.

An explanation of the study’s target, ensuring voluntariness and secret, and extending the contact information of the major investigator were all on the cover page of the questionnaire. The questionnaire was split into two sections. The first one was about sociodemographic features. The second one contained nine multiple-choice questions concerning knowledge in terms of the safe utilization of dental imaging radiographs for pregnant women (Additional file 1: Appendix A). An additional file includes all questions and options. The overall potential score for the awareness questions was 9 points, and correct answers were given a score of 1, while incorrect answers were given a score of 0. The level of knowledge was categorized by the number of correct answers (poor: 0–3; fair: 4–6; good: 7–9).

### Study variables

Participants' ages (30, 30–39, 40–49, and 50), marital status (married or single), and level of education (less than high school, high school graduate, college/university, post-graduate studies (MSc and PhD) were all demographic features. The participants were also asked if they worked or studied in the radiation sciences (yes or no), the medical field (yes or no), or any other field (yes or no).

### Statistical analysis

Statistical Package for the Social Sciences (IBM SPSS) software (statistics version 26) was used as statistical analysis software. Furthermore, univariate analyses like percentages and frequencies were applied to determine the properties of the participants and their perspective and awareness. On the other hand, bivariate analyses were done to compare the awareness of women against the features of the research study sample. Multi-nominal logistic retraction analysis was done to assess the associations between the various predictors and levels of awareness. Independent variables were chosen from previous literature studies [23]. The marital status, age, education level, and whether the participant was working or studying in the medical field were adjusted in this study. However, the significance level was set at *P* < 0.05.

## Results

This research study included 984 women with no lost information on the study elements. The majority (64.7%) of the participant’s women were aged < 30 years, and they were single and married. More than two-thirds of the participants had a bachelor's degree, and the majority were studying or working in the medical field. Only 16.5% of the participants had good knowledge, whereas 16.7% of them had insufficient knowledge concerning dental imaging during pregnancy (Table 1). Among the respondents of this study, several participants were not perceptive of the situation of the radiation safety measures throughout dental imaging. Most of the respondents mentioned that pregnant women should tell the radiologist about that. While 25.4% announced that pregnant women would be able to have dental radiographs in any trimester of their pregnancy period. The majority assured us that the aprons made of two layers are required. Moreover, 61.4% of the respondents reported that the dental imaging radiograph with a single dose was less than the normal background radiation. In terms of the sort of radiographs that can be obtained throughout the pregnancy period, 46.2% and 34.6% reported that CBCT and panoramic radiographs are not contraindicated, respectively. 71.4% of the participants mentioned that the fetal deformity and cancer risk that is caused by radiation exposure is very low (Table 2).

**Table 1:** Characteristics of the study population (n = 984)

	Frequency	Percent
*Age*		
< 30	637	64.7
30–39 years	221	22.5
40–49 years	89	9.0
≥ 50	37	3.8
*Marital*		
Single	495	50.3
Married	489	49.7
*Education level*		
Less than high school	2	0.2
High school graduate	75	7.6
Collage	739	75.1
Master degree	147	14.9
Ph. degree	21	2.1
*Sectors*		
Technician or radiologist	198	20.1
Medical field	414	42.1
Others	372	37.8
*Level of knowledge*		
Good (score 7–9)	162	16.5
Fair (score 4–6)	658	66.8
Poor (score 0–3)	163	16.7

**Table 2:** Overall Knowledge about the precautionary measures of taking dental radiographs during pregnancy

Knowledge items	Frequency	Percent	Listed statements are true (T) or false (F)
Pregnant women should inform the radiologist if she is pregnant or expecting	930	94.5	T
Pregnant women can take radiographs at any trimester	250	25.4	T
Pregnant women should wear a lead apron and thyroid collar while taking a dental radiograph	792	80.5	T
The radiation dose during pregnancy is less than the usual dose	604	61.4	F
Pregnant women can take CBCT	455	46.2	T
The risk of fetal malformation due to radiation exposure is very low?	481	48.9	T
The risk of cancer among infants due to radiation exposure is very low?	703	71.4	T
Does panoramic imaging (radiography of the teeth) put the fetus at risk (for example, miscarriage or malformations)?	455	46.2	F
Pregnant women can take a panoramic radiograph	340	34.6	T

The multinomial logistic retraction analysis was managed to carry out the union of the predictors and the level of knowledge they have. The awareness questions evaluated the knowledge of pregnant women relating to the protection and misconceptions of having diagnostic dental radiographs during the pregnancy period. The significant predictor linked to the level of participant’s knowledge was with respondents who were studying or working in the medical field (odds ratio [OR]: 0.8; *P* < 0.05). Furthermore, studying or working in the medical field does increase the likelihood of having more excellent knowledge about the caution of having dental radiographs throughout pregnancy. However, the marital status of participants, education level, and age were not linked with the level of knowledge (Table 3).

**Table 3:** Multinomial logistic regression showing predictors of knowledge level

Characteristics	Odds ratio (OR)	95% confidence interval	*P*-value	
Age	< 30	Ref	Ref	0.03*
	30–39 years	1.0	0.8–1.3	
	40–49 years	1.3	0.9–1.8	
	≥ 50	1.4	0.9–2.3	
Marital	Single	Ref	Ref	0.551
	Married	0.9	0.8–1.1	
Education level	Less than high school	0.9	0.1–7.6	0.597
	High school graduate	0.7	0.4–1.5	
	Collage	0.7	0.4–1.3	
	Master degree	0.8	0.4–1.5	
	Ph. Degree	Ref	Ref	
Sectors	Technician or radiologist	Ref	Ref	0.393
	Medical field	0.8	0.7–1.1	
	Others	1.0	0.8–1.4	

*P < 0.05

## Discussion

Dental radiography is an essential part of dentistry as it is a crucial diagnostic tool and image of the human teeth that the dentist uses to evaluate the oral health. Previously, dental imaging was prevented during pregnancy, particularly during the first trimester (the earliest phase of pregnancy), to keep the developing fetus safe. As a result, oral health may be compromised during pregnancy, and medical imaging examinations for accurate diagnosis and management of various dental conditions may be required. Pregnant women are typically hesitant to have dental radiography taken, which may delay necessary treatment and negatively impact the health of both the fetus and the mother.

When the measurements of radiation protection are applied during dental imaging for pregnant women, the imaging will be safe with no risks [24]. Radiation doses can be largely minimized by different measures [2]. The best way to decrease radiation doses by a factor of 10 is by applying F-speed film (digital sensors) in integration with rectangular collimation for bitewing and full mouth radiographs [25].

A small percentage of the participants had knowledge that dental radiographs can be obtained over any trimester but with the application of radiation protection techniques. Almost two-thirds of the respondents reported that it was prevented in all trimester pregnancies. Most participants thought that CBCT and panoramic radiographs were avoided during pregnancy. Less than 19.5% of the participants were confused about the radiation protection applications that should be applied during dental radiographs. The majority of participants had mistakes or misconceptions, for example, about the presence of a particular lead apron designed for pregnant patients or that double layers of lead aprons are required. Although the risks from dental imaging are low to nonexistent even without the use of fetal and gonadal lead shields [26], it is recommended that even if the lead shielding is unimportant, it provides the patient with comfort and a sense of protection [26–28].

A potential justification about the awareness of risks from dental imaging is the shortage of public radiation knowledge programs. Moreover, it is possible that patients are not knowledgeable regarding radiation protection and hazards from their dentists. A previous research study by Al Faleh et al. [23] mentioned that about 40% of the patients were not informed regarding the radiation risks by their treating dental practitioners. Most of the patients did not in the least inquire about protection measures before taking an imaging. Also, patients’ loss of information could be a throwback to incomplete knowledge between dentists. A comprehensive literature review mentioned that there is global concern about dentists’ knowledge in terms of dental radiographs over pregnancy. Various research and review studies have reported that the awareness of dentists, dental students, and interns about radiation and its safety is insufficient [16, 18, 29–31]. Another research study, Aboalshamat et al., reported that 67% of dentists believed periapical imaging was safe only over the second trimester (spanning week 14 to week 27). 69% of the participants underwent panoramic imaging while pregnant [30].

However, 2% of dental practitioners knew that a dental radiograph was safe in all trimesters of pregnancy, whereas 44% believed it was unsafe in any trimester [29]. Moreover, a research study in Jordan discovered that more than half of the Jordanian dentists believed that panoramic radiographs were avoided during pregnancy, while less than 33% did not know if they were safe or not [18]. Llea et al. [16] pointed out that more than two-thirds of dental practitioners would ask for dental imaging only for emergency needs. It may be that dental professionals have no knowledge about the considerable dose reduction linked with digital imaging compared to conventional film. A shortage of information could cause great anxiety for both dental practitioners and pregnant women searching for dental treatment during pregnancy.

Information regarding radiation doses from dental radiographs proportional to the background radiation dose was insufficient. The comparison in doses between the periapical imaging and the background radiation was not assured among most participants. As a comparison, the dose of single bitewing imaging obtained with a suitable collimator (rectangular collimator) and photostimulable plate is less than the dose of one day of background radiation [25]. The National Council on Radiation Protection and Measurements mentioned that the dose for fetal from full-mouth intraoral imaging is 4–6 times less than the dose of background radiation during a mother's pregnancy [2].

Regarding congenital deformities, a very small percentage of participants knew that such hazards were not linked to dental radiographs, while more than half of the respondents knew that the hazard of radiation-induced congenital deformities from dental imaging was very high. In a similar way, Razi et al. [32] reported that only 28% of dentists realized that radiation exposures from diagnostic radiographs do not affect congenital deformities or fetal mental problems. Concerning fetal deformities, the International Commission for Radiation Protection states that the fetal absorbed dose must be equal to or greater than the threshold dose of 100–200 mGy. This is quite more than the fetal absorbed exposures from diagnostic imaging, in addition to nuclear imaging. In studies applied to humans and animals, there is no proof that the range of radiation dose from diagnostic imaging (i.e., less than 50 mGy) is related to a raised hazard of teratogenic impacts [24, 33, 34].

The danger of infant cancer is complex to estimate from low-level doses, such as dental radiographs [25]. In this study, participants lacked assurance regarding the oncogenic hazards of dental radiographs. The remaining, nearly one-third of the participants, thought that the hazard was large; less than one-third thought that the hazard was low; and the remaining nearly one-third thought the hazard did not exist at all. The note that radiation can lead to cancer is obtained from previous studies of the atomic bombs that happened at Hiroshima and Nagasaki and other group studies. Furthermore, epidemiologic studies have not been successful in establishing a link between the dose and cancerogenic effects [25, 33, 34]. The fetal head dose from a single computer tomography (CT) scan has been measured in the range of 0–0.005 mGy [33, 34].

The limitations of this study are diverse for several reasons, such as the absence of revealing the causality; applying snowball sampling, which weakens the potency to popularize the results; this study may be able to self-chosen bias due to the kind of recruiting participants that may venture both internal and external legality.

The future recommendations of this research study are guaranteed to evaluate obstetricians’ view of radiation dose and hazard linked with different dental radiographs during pregnancy. In addition, further research will be proposed to estimate the efficacy of an educational intervention tailored to teach people about radiation exposure and danger to their advantage.

## Conclusions

Our research study has shown that there is a shortage of information in the Jordanian population concerning the protection of dental radiographs during pregnancy. Women’s views of the hazard from dental radiographs are unexpectedly high. This incorrect understanding could lead to worry, anxiety, and delays in important dental treatment. Because women's knowledge has an immediate impact on their attitude and behavior toward dental protection, it is critical to establish social awareness campaigns that aim to educate our community about radiation doses, protection, and important safety measures. Dental professionals should educate pregnant patients in terms of the protection of dental radiographs throughout pregnancy and demonstrate their advantages and hazards. The direct effect of this misconception will be on behavior toward seeking dental care. Thus, social knowledge initiatives aimed at telling our community about safety, radiation exposure, and the desired protection measures are important.

## Supplementary Information

**Additional file 1: Appendix A.** Full survey of this research study.

## Data Availability

All data generated or analysed during this study are included in this published article [and its Additional file].

## Abbreviations

CBCT: Cone-beam computed tomography
PI: Panoramic images
ALARA: As low as reasonably achievable
AAPM: American association of physicists in medicine
