# Supplementary material for: Jordanian women’s (studying or working in medical fields) awareness in terms of the use of dental imaging during pregnancy
Source: BMC Oral Health. 2022 Sep 24;22:427. doi: 10.1186/s12903-022-02459-w (PMC9508717; doi:10.1186/s12903-022-02459-w)
Supplement: Supplementary file 1 — Additional file 1: Appendix A. Full survey of this research study. [file 12903_2022_2459_MOESM1_ESM.docx]

Appendix A: full survey of this research study.

| **Section 1: General Information** |
| --- |
| Evaluation of Jordanian Women’s awareness regarding the use of dental imaging during pregnancy  Date……………………..  Please circle the most appropriate response.  1. Marital Status: a) Single b) Married  2. Please write your AGE:………………………….  3. Education: a) School b) Collage/University  4. Work or study in the Medical field? a) Yes b) No  5. Level of knowledge about dental imaging during pregnancy:  a) Poor b) Fair c) Good |

| **Section 2: Knowledge about the precautionary measures of taking dental radiographs during pregnancy** |
| --- |
| 1. Pregnant women should inform the radiologist if she is pregnant or expecting?  2. Pregnant women can take radiographs at any trimester?  3. Pregnant women should wear a lead apron and thyroid collar while taking a dental radiograph?  4. The radiation dose during pregnancy is less than the usual dose?  5. Intraoral films should be held by the film holder?  6. Pregnant women can take a panoramic radiograph?  7. Pregnant women can take CBCT?  8. The risk of cancer among infants due to radiation exposure is very low?  9. The risk of fetal malformation due to radiation exposure is very low? |
